# The Effects of Low and High Levels of Sadness on Scope of Attention: An ERP Study

**DOI:** 10.3389/fpsyg.2018.02397

**Published:** 2018-11-27

**Authors:** Hailu Wang, Ying Chen, Qin Zhang

**Affiliations:** Beijing Key Laboratory of Learning and Cognition, Department of Psychology, Capital Normal University, Beijing, China

**Keywords:** sadness, attention scope, global–local attention, emotion, event-related potential

## Abstract

Sadness has inconsistent effects on the scope of attention. These differing effects may be attributed to different levels of sadness induced in different experiments. Low levels of sadness can expand the scope of attention, but high levels can narrow it. In this study, we recruited 42 college students and induced different levels of sadness in them by having them view sad images continuously, and then we assigned them Navon’s letter task. The results showed that among participants with local-processing bias, those at lower levels of sadness were slower to identify small letters than were those at high levels of sadness and control condition (watching neutral images). Event-related potential (ERP) results showed that low-sadness participants put more attention resources toward processing large letters (global stimuli). They showed increased amplitude of the P1 component compared with high-sadness participants and participants at control condition. These results suggested that different levels of sadness had different effects on attention scope: low levels of sadness extended the scope but as sadness increased, this extension disappeared. This influence pattern mainly occurred in the early stages of visual processing.

## Introduction

To date, a great deal of research has focused on the interaction between emotion and attention (Srinivasan and Gupta, 2010, 2011; Srinivasan and Hanif, 2010; Srinivasan, 2015; Gupta et al., 2016). However, there has been little research on the effects of sadness on attention scope. In recent years, some studies have explored sadness’s impact in this area, but the findings have been inconsistent. Some studies have found that sadness expanded participants’ attention scope, while others have found that it narrowed the scope. A good deal of research aimed at the effects of emotion on attention scope has found that when people are in a positive emotional state, the scope of attention is expanded (Fredrickson, 1998, 2001); but when they are in a negative emotional state, the scope is narrowed (Finucane, 2011).

Sadness is a typical negative emotion. Per the abovementioned conclusions, the scope of attention is narrow when people feel sad, since they will focus their attention more on details rather than on global information. This has been proven by the results of some studies (Wyer et al., 1999; Clore and Gasper, 2000; Clore et al., 2001), in which cues from sad moods may have been experienced as promoting attention to local information (Gasper and Clore, 2002). Recent studies have indicated that participants who were sad or mildly to moderately depressed showed less flanker interference than participants who were neither sad nor depressed (Melcher et al., 2012; Bellaera and von Mühlenen, 2016). In these studies, the target stimulus was an arrow that appeared in the middle of the screen, pointing either left or right; when the stimulus presented, so did interference arrows on either side of the target arrow, pointing either in the same or the opposite direction. The decrease in interference showed that participants had focused on the target arrows, paying less attention to disturbance stimuli; this indicated a narrowed scope of attention.

However, some research has shown that sadness expands attention scope. In these studies, sadness was induced by watching pictures and then asked participants to complete the Navon letters task (Navon, 1977). The results of a study by Gable and Harmon-Jones (2010) indicated that sadness caused attentional broadening: participants responded more slowly to small letters (local targets) after viewing sad images than after viewing neutral ones. In a study by Zhang and Zhou (2013), participants responded faster to large letters (global targets) after viewing sad images than after viewing neutral ones. These results indicated that sad affective states can extend attention scope. In addition, Finucane et al. (2010) reported that executive control (flanker task) of sad and happy groups were not significantly different compared with neutral group. A recent study found that sadness had no significant effect on the reaction time of global/local stimuli (von Mühlenen et al., 2018).

The reasons for these discrepancies in findings are not clear. One possible reason is that different experimental procedures evoke different subjective levels of sadness. Some of the experiments evoked lower levels, while others evoked higher ones. The difference in degree of sadness experienced by participants led to different effects on attention scope. A low level of sadness can expand the scope of attention, but when this level rises, the expansion effect reverses, resulting in a narrowing of attention scope. In particular, the study by Bellaera and von Mühlenen (2016), which used a special procedure to induce emotion, typically induced prolonged and more-intense emotional states. Other studies induced different emotional states that were less intense and did not last as long (Carvalho et al., 2012; Bellaera and von Mühlenen, 2016). However, this inference needs to be validated.

In our study, we used a holistic design in one experiment to induce different levels of sadness and to quantitatively explore the influence of sadness on attention scope. We used an event-related potential (ERP) technique to examine the neural mechanisms underlying the effects of different sadness levels on scope of attention. We focused only on 3 components of ERP. Early visual-processing components (P1) are sensitive to physical properties of stimuli, reflecting early perceptual processing with the visual striatum cortex (Olofsson et al., 2008). Studies have suggested that an increase of P1 amplitude reflects the capture of attentional resources (Delplanque et al., 2004; Olofsson et al., 2008). The N2 component, a negative wave in the frontal area peaking 200–350 ms after stimulus onset, is considered to reflect interference inhibition or conflict control. Incompatible stimuli elicit the central or frontocentral N2 component; inconsistent targets evoke larger N2 amplitude than do consistent targets (Han et al., 1997, 1998; Folstein and Petten, 2008). The P3 component, the positive wave that peaks around 400 ms, is related to attention monitoring and working memory and reflects the allocation of limited attention resources; inconsistent targets decrease P3 amplitude to local figures (Han et al., 1997; Linden, 2005; Hajcak et al., 2009).

In sum, a primary purpose of this study was to test whether varying degrees of sadness had different effects on attention scope. Given previous studies (Gable and Harmon-Jones, 2010; Zhang and Zhou, 2013; Bellaera and von Mühlenen, 2016), we expected to find that a low level of sadness extended attention scope while a high level narrowed it. Another important purpose of the study was to use ERP for the first time to explore the neurophysiological mechanisms behind these effects and to learn how different degrees of sadness affected different stages of cognitive processing by analyzing differences in mean amplitude for the P1, N2, and P3 components.

## Materials and Methods

### Participants

Forty-two students (9 males, 33 females; mean age 23.9 years, range 18–29 years) from Capital Normal University, Beijing, China, participated in the experiment for ¥90 each. All participants were right-handed, with normal vision or corrected visual acuity and no visual impairments (color blindness or color weakness). Before starting the experiment, each participant read and signed an informed-consent form. This study was approved by the Psychology Ethics Committee of Capital Normal University.

### Sadness Induction

**FIGURE 1:**
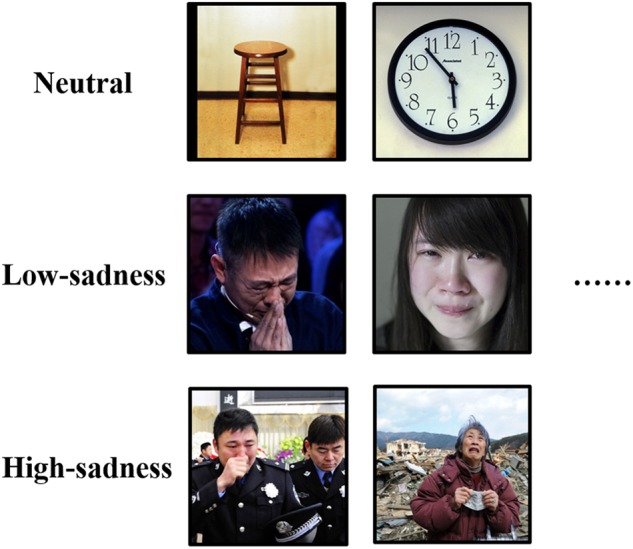
Sample for pictures of each block in the experiment.

In this experiment, we used 120 images to induce different emotional states in participants. To reduce time spent searching for images, we selected 40 neutral images from the International Affective Picture System (Lang et al., 1999) and used Google search engine to search and download 80 other sad images on the Internet. All sad images were downloaded for free, and we used them only to arouse the emotions of participants, not for other purposes. The resolution size of all images was 1024 × 768. All neutral images depicted common items such as cups or tables. Sad images were either low sadness or high sadness (40 for each type). Low-sadness images depicted people who were weeping due to having just experienced setbacks or sad memories. High-sadness images depicted people crying due to having just experienced major disasters, such as losing relatives and homes in an earthquake or friends and partners in an accident. Another difference between the low- and high-sadness images was that the latter included wider scenes, such as a house that had collapsed after an earthquake or the family members that had been lost, while the low-sadness images did not include such specific scenes (Figure 1). To avoid feelings of fatigue or disgust, participants viewing neutral images were asked to simply observe the objects in the picture. Before showing them either low-sadness or high-sadness images, we briefly introduced the images by asking participants to comprehend the contents of the images seriously and to feel the emotions of the people depicted. The brief introduction to low-sadness images was, “The people in these images have just experienced setbacks or are recalling sad memories, so they are shedding tears of sorrow.” The introduction to high-sadness images was, “These pictures were taken at the actual scenes of disasters or accidents. The people in the images have just experienced a sudden disaster, and they have lost control and are crying out loud at the loss of their homes and relatives.”

### Attention Scope Test

**FIGURE 2:**
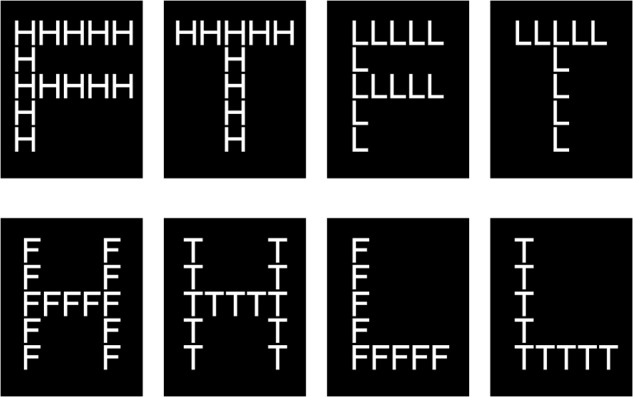
Navon letters used in the experiment.

We measured scope of attention using the Navon (1977) global–local letter task, an objective measure of attentional breadth used in many past studies (Kimchi, 1992; Yovel et al., 2005; Gable and Harmon-Jones, 2010). In our experiment, we displayed 4 capital letters—H, L, F, and T—composed of horizontal and vertical lines made up of smaller, different capital letters. For example, the large capital H was composed of small capital Fs. This experiment contained a total of 8 stimuli (Figure 2). When the stimulus contained the letter H, the participant was told to press the left mouse button; when it contained the letter L, whether large or small, the participant was told to press the right mouse button. The button was to be pressed “as quickly as possible.” Pictures consisting of a large H or L composed of small Fs or Ts were defined as global targets, and those consisting of a large F or T composed of small Hs or Ls were defined as local targets. Participants’ responses to large and to small letters, respectively, reflected global and local levels of attention scope. If the response to global targets was faster than that to local targets, the scope of attention was wide; if the converse was true, the scope was relatively narrow. In each stimulus, the height angle of small letters was 0.72° and the width angle 0.53°, while the height angle of large letters was 3.58° and the width angle 2.63°. The background color of all stimuli was black (RGB:0.0.0) and the foreground color white (RGB:256.256.256).

### Procedure

We divided the experiment into 3 blocks by type of emotion: neutral, low-sadness and high-sadness images. We used the Latin square method to balance the block sequence between participants. For each emotional condition (neutral, low-sadness and high-sadness), we used pseudorandom processing to dictate the order of large and small letters. Before the formal experiment, we trained participants on the task in the attention scope test. After completing practice trials for attention test training, all participants reported that they were familiar with the experimental requirements, and the accuracy rate was > 96.7%.

**FIGURE 3:**
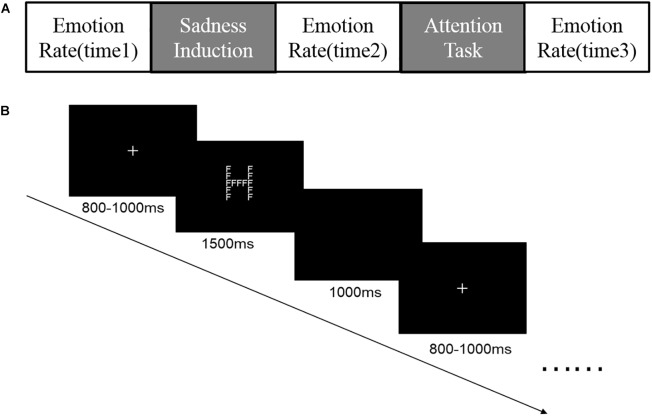
**(A)** Five phases of each block in the experiment; **(B)** Procedure for the Navon letters task.

Each block consisted of 5 phases (Figure 3): initial emotional rate (time1), sadness induction, secondary emotional rate (time2), Navon letter task and tertiary emotional rate (time3). In order to ensure effective induction of different degrees of sadness, we measured participants’ emotional states during all the phase of emotional rate. We used 4 typical negative emotional adjectives—angry, sad, disgusted, and afraid—to measure emotional state. Participants were asked to rate their emotional states on a scale of 1–5 (1: do not experience this feeling; 5: strongly experience this feeling). During the sadness induction phase, all images were played automatically, and each was presented for 6 s. In the neutral block, participants were asked to simply observe the image, but for sad blocks they were asked to carefully look at what was in the image and understand the emotions of the people depicted. During the Navon letter task phase, each trial began with a white cross fixed at the center of a black screen for 800–1000 ms, followed by a Navon letter with white coloring for 1500 ms. The participants had to respond to the letter in 1500 ms, after which it disappeared and a blank screen was shown for 1000 ms (Figure 3). There were 80 trials per block; trials were presented in pseudorandom order.

The experiment was conducted in a soundproof, electromagnetically shielded room with mild light. We used a 17-inch cathode ray tube (CRT) computer screen with a resolution of 1024 × 768 and a refresh rate of 100 Hz to present all stimuli. The experimental program was written with E-Prime software version 2.0 (Psychology Software Tools, Inc., Sharpsburg, PA, United States).

### Electroencephalogram (EEG) Recordings and Data Processing

We recorded EEGs using a Neuroscan SynAmps2 amplifier (Compumedics Neuroscan, Sterling, VA, United States), with 64 Ag/AgCl electrodes positioned in an electrode cap according to the International 10–20 system. Vertical and horizontal electrooculographs were recorded from electrodes located below and above the left eye and at the outer canthus of each eye. A left-mastoid reference electrode was used online, and the reference was changed offline to the average of the left- and right-mastoid recordings. We filtered EEG signals with a band pass of 0.05–40 Hz and sample data rate of 500 Hz. Electrode impedances were kept < 5 KΩ. Incorrect response trials and artifacts exceeding ± 75 μV were excluded from ERP analysis. Each averaged epoch of the attention task phase lasted 1700 ms, including 200 ms before onset of the Navon letter stimulus to serve as a baseline.

**FIGURE 4:**
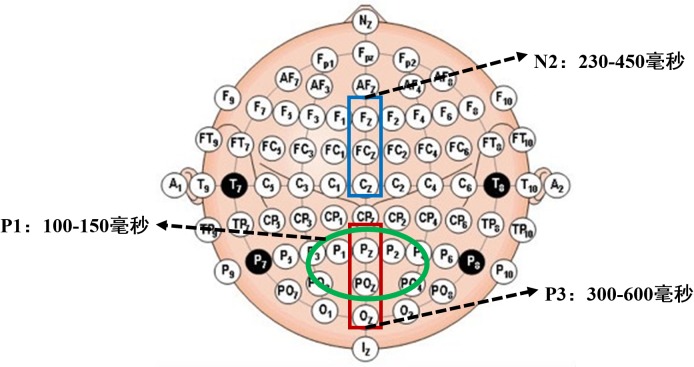
Time-windows and scalps of P1, N2, and P3.

According to our preliminary study, we selected P1, N2, and P3 for analysis. Six scalp electrodes, P1, P2, Pz, PO3, PO4, and POz, were selected to analyze mean amplitude of the P1 component. We selected Fz, FCz, and Cz to analyze mean amplitude of N2; and Pz, POz, and Oz to analyze that of P3. Time windows and scalp electrodes for all ERP components are shown in Figure 4.

### Design

This experiment employed a 2 × 3, two-factor, within-subject design. We used 2 independent variables, attention scope and degree of sadness. Dependent variables were RT and the ERPs of participants when performing attention tasks. All participants were required to finish attentional tasks under all emotional conditions.

### Data Analysis

The core purpose of this study was to explore the influence of different levels of sadness on attention scope. It used a block design in which each block corresponded to an emotional state induced by presenting images in succession, after which we tested attention scope. Each block lasted about 10–15 min; the duration of emotional induction and of the attention task took up about half of this time span. After completing a block, participants rested for 5–10 min. The entire experiment lasted about 40–60 min. After we had effectively induced the desired emotional state in participants, we analyzed its effect by studying the accuracy rate and RT of the attention task (Navon letters task) and the average amplitude of ERP components evoked during the phase of this task.

One participant did not complete the experiment, and the behavioral data of another (who was in a state of low sadness) was partially lost and thus could not be used for analysis. In this experiment, the accuracy rate for all participants during the Navon letter task was > 97.8%.

## Results

### Behavioral Grouping

Interestingly, in neutral conditions (baseline), roughly half of the participants (21) responded faster to local stimuli (small letters) than to global stimuli (large letters), while the other half (19) were the opposite. Considering that potential attention bias would influence the effects of emotion on attention scope, it was necessary to divide the participants into 2 groups, local-faster group (LFG) and global-faster group (GFG), and then analyze the data separately. We conducted behavioral and ERP data analysis independently in the 2 groups.

**FIGURE 5:**
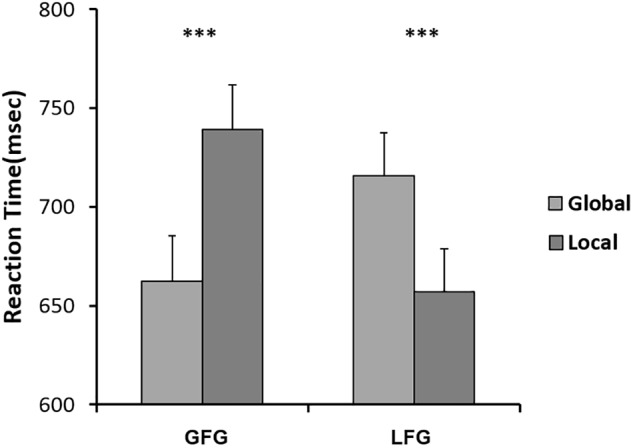
Mean RTs for Navon letters on GFG and LFG, respectively. Error bars indicate standard errors. ^∗∗∗^*p* < 0.001.

We submitted RTs obtained from correct-response trials to 2 (group: LFG, GFG) × 2 (stimulus: global, local) repeated analysis of variance (ANOVA) measures, and we found an interactive effect: *F*(1,38) = 64.296, *P* < 0.001, η^2^ = 0.629. This pattern is shown in Figure 5.

### Emotion Rate

The mean value of emotional rate for all participants is shown in Table 1. Repeated ANOVA measures [4 (emotional types: fear, sadness, disgust, anger) × 3 (degree of sadness: neutral, low, high) × 3 (rate time: time1, time2, time3)] showed that all main effects and interaction effects were significant (*P* < 0.001). Further analysis found that after participants viewed neutral images, there was no significant difference between the 4 emotional types or the 3 rate times (*P* > 0.5). After participants viewed both low- and high-sadness images, sadness was significantly higher than other emotional states (fear, disgust, and anger) at time2 (*P* < 0.001). Most importantly, after viewing both high- and low-sadness images (time2), all participants evinced more sadness than they did after viewing neutral images (*P* < 0.001); and compared with low-sadness images, high-sadness images induced higher levels of sadness (*P* < 0.001). The GFG and LFG groups showed the same effects. The above analysis indicated that participants experienced a greater sense of sadness after viewing sad images, and the sadness was more intense after viewing high-sadness images than after viewing low-sadness images.

**Table 1:** Means of emotion rating (all participants).

Emotion types	Neutral			Low-sadness			High-sadness		
	time1	time2	time3	time1	time2	time3	time1	time2	time3
fear	1.200	1.175	1.075	1.050	1.700	1.175	1.250	2.125	1.475
sad	1.400	1.175	1.200	1.175	2.675	1.675	1.250	3.600	2.100
disgust	1.125	1.150	1.250	1.050	1.625	1.475	1.100	1.675	1.650
anger	1.125	1.100	1.100	1.100	1.425	1.275	1.125	1.650	1.400

### Behavioral Results

**FIGURE 6:**
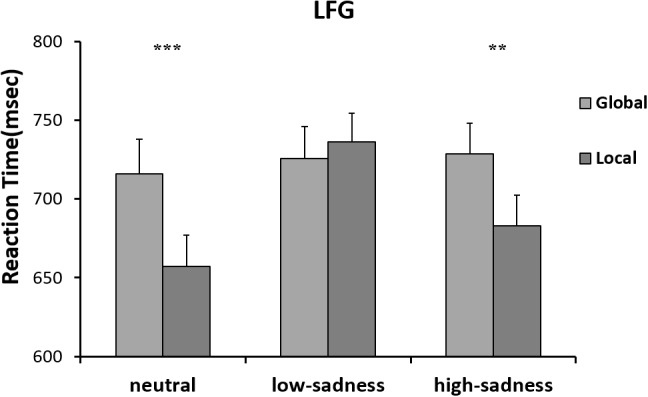
Mean RTs for the Navon letters in LFG. ^∗∗^*p* < 0.01, ^∗∗∗^*p* < 0.001.

**FIGURE 7:**
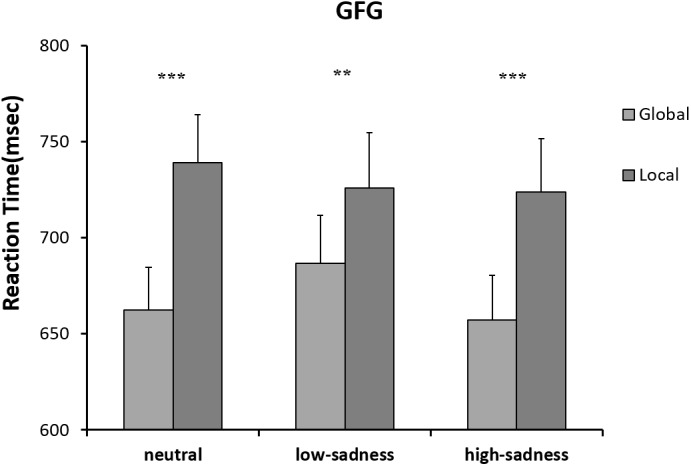
Mean RTs for the Navon letters in GFG. ^∗∗^*p* < 0.01, ^∗∗∗^*p* < 0.001.

The results of the 2 (attention scope: global, local) × 3 (sadness degree: neutral, low, high) repeated ANOVA measures showed no significant interaction effect or any main effects on RTs of any participants. For the LFG, there was a significant main effect on attention scope [*F*(1,20) = 8.3, *P* = 0.009, η^2^ = 0.293]. Compared with those to global stimuli, mean RTs to local stimuli were shorter. The interaction effect was significant [*F(*2,40) = 11.148, *P* < 0.001, η^2^ = 0.358]. Further analyses showed that after LFG participants viewed neutral and high-sadness images, mean RTs to local stimuli were significantly shorter than those to global stimuli (*P* < 0.01), but there was no significant difference after viewing low-sadness images (Figure 6). Mean RTs to local stimuli after viewing low-sadness images were longer, compared with mean RTs after viewing neutral and high-sadness images (*P* < 0.05). For the GFG, 2 (attention scope: global, local) × 3 (sadness degree: neutral, low, high) repeated ANOVA measures showed a significant difference only in the main effect of attention scope [*F*(1,18) = 45.872, *P* < 0.001, η^2^ = 0.71; Figure 7].

### ERP Results

Based on visual inspection of grand average waveforms, we computed mean amplitudes at time windows 100–150 ms (P1), 230–450 ms (N2), and 300–600 ms (P3) for each participant and emotional state. For ANOVA measures, we selected 12 electrodes: P1, P2, Pz, PO3, PO4, and POz for component P1; Fz, FCz, and Cz for N2; and Pz, POz, and Oz for P3. To investigate the effects of different degrees of sadness, we carried out 2 (sadness degree: neutral, low, high) × 3 (attention scope: global stimuli, local stimuli) repeated ANOVA measures in 3 time windows.

#### P1 (100–150 ms)

**FIGURE 8:**
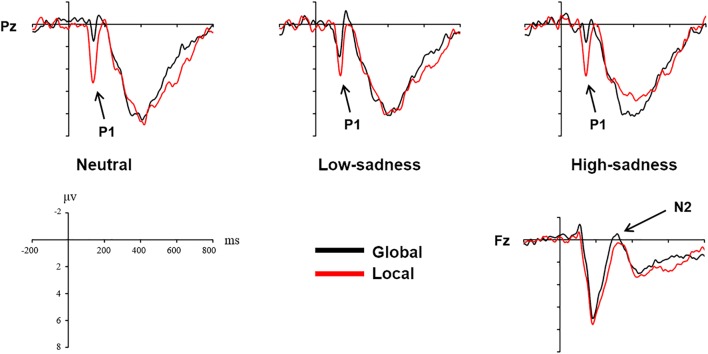
Grand mean amplitudes of P1 and N2 across stimulus type (LFG).

**FIGURE 9:**
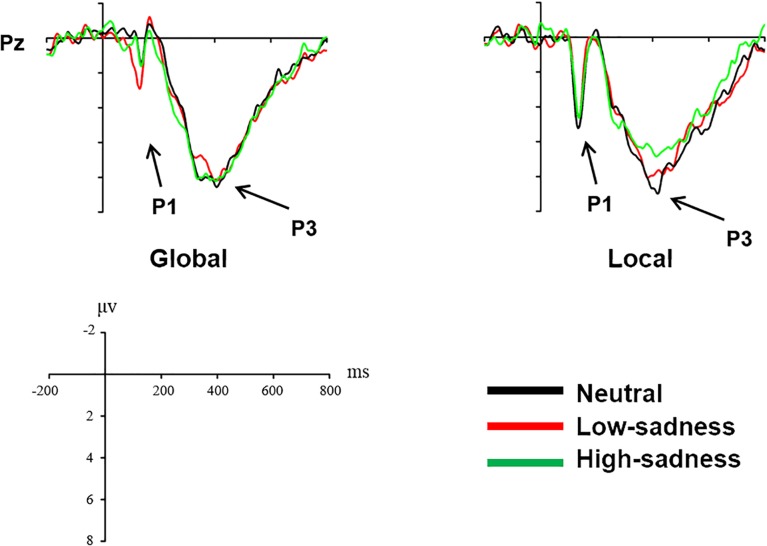
Grand mean amplitudes of P1 and P3 across sadness degree (LFG).

**FIGURE 10:**
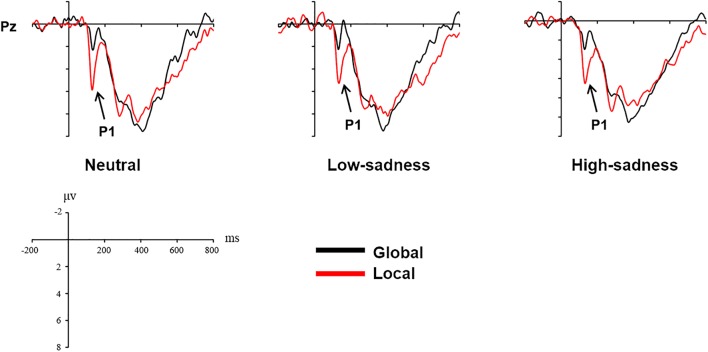
Grand mean amplitudes of P1 across stimulus type (GFG).

For LFG participants, the analysis revealed a significant main effect on attention scope [*F*(1,20) = 20.98, *P* < 0.001, η^2^ = 0.512] and a significant interaction effect [*F*(2,40) = 6.759, *P* = 0.003, η^2^ = 0.253]. Local stimuli evoked larger positive amplitudes than did global stimuli. Further analysis revealed that after participants viewed neutral and high-sadness images, local stimuli evoked larger positive amplitudes than did global stimuli (*P* < 0.05), but this significance disappeared after participants viewed low-sadness images (*P* > 0.1; Figure 8). More importantly, positive amplitudes evoked by global stimuli were significantly larger after participants viewed low-sadness images compared with neutral and high-sadness images (*P* < 0.05; Figure 9). For GFG participants, the analysis revealed a significant main effect on attention scope only [*F*(1,19) = 24.63, *P* < 0.001, η^2^ = 0.565], and no other main or interaction effects were found. Local stimuli evoked larger positive amplitudes than did global stimuli at high, low and neutral degrees of sadness (Figure 10).

#### N2 (230–450 ms)

For LFG participants, the analysis revealed a significant main effect on attention scope only [*F*(1,20) = 4.94, *P* = 0.038, η^2^ = 0.198]. Compared with local stimuli, global stimuli evoked larger negative N2 amplitudes (Figure 8). No other main or interaction effects were found for the GFG or for all participants.

#### P3 (300–600 ms)

For LFG participants, the analysis revealed a significant interaction effect [*F*(2,40) = 3.24, *P* = 0.05, η^2^ = 0.14]. Further analysis revealed that the positive amplitudes evoked by local stimuli were smaller after participants viewed high-sadness images compared with neutral images (*P* < 0.05), but there was no significant difference in amplitudes evoked by global stimuli (Figure 9). No other main or interaction effects were found for the GFG or for all participants.

## Discussion

This is the first study to explore the effects of varying degrees of sadness on attention scope in one experiment. Induction of sadness is the basis of such research. Previous studies induced sadness in various ways, mostly using images or film, although some used music. In our experiment, to effectively induce different levels of sadness, we adopted the method of continuously displaying images with brief introductions attached. We measured 3 phases of sadness, focusing mainly on the first (time1) and second (time2) measurements. Data analysis showed that, after participants viewed neutral images, there was no difference between any of the emotions measured at each time. After viewing images at time2, participants experienced mostly sadness compared with other emotions; compared with neutral images, low-sadness images induced a moderate degree of sadness (score 2.5–2.8), while high-sadness images induced more-intense sadness (score 3.4–3.7). Most importantly, the difference was significant between high and low degrees of sadness. Therefore, it can be said that the experiment effectively induced different levels of sadness in participants, an effect that remained valid after grouping. However, data analysis results showed that on the condition of sadness, fear and disgust levels rose significantly after viewed images. This might be because the high-sadness images contained content related to death. While these emotions were not as strong as sadness, their effects need further exploration.

Unlike in previous studies (Gable and Harmon-Jones, 2010; Zhang and Zhou, 2013), participants in our study showed significant differences in response to global/local stimuli, which may indicate a attentional bias. Half of participants in our experiment responded faster to small letters than to large letters, which might demonstrate local bias in response to Navon letters. While other participants showed the opposite pattern. If this bias does exist in people’s attention scope, it is likely to affect the effects of sadness on attention.

For LFG participants, the effect of sadness degree was significant. After viewing low-sadness images, participants’ response to local stimuli was slow, resulting in disappearance of the local bias shown after participants had viewed neutral images. This result was partially consistent with previous studies by Gable et al. (Gable and Harmon-Jones, 2010). However, when LFG participants experienced a high level of sadness, the local bias reappeared, and response to local stimuli was faster than that of participants experiencing a low level of sadness. This proved our previous inference: different degrees of sadness had different influence patterns on scope of attention. Inconsistencies in previous studies were indeed caused by varying degrees of sadness induced by different experimental approaches. However, no similar patterns were found for GFG participants, who showed no differences in effects due to different degrees of sadness. Global bias remained unchanged for neutral, low or high sadness, probably because GFG participants reached the maximum global effect in the experiment, so low sadness could not further extend their scope of attention. In summary, the behavioral data showed that the influence of sadness on attention scope varied with degree of sadness; moreover, the attentional bias of individual participants had a moderating effect on this influence. The inconsistent findings of previous studies (von Mühlenen et al., 2018) may be due to the lack of discrimination in different levels of sadness.

The P1 component in the posterior part of the scalp is an early component associated with visual perception. The oscillograms of all participants showed that the P1 amplitudes evoked by local stimuli were significantly greater than those evoked by global stimuli, and this was consistent across all levels of sadness. Most importantly, in group LFG, mean P1 amplitude differed by level of sadness. After participants had viewed neutral and high-sadness images, local stimuli evoked significant differences in P1 amplitude compared with global stimuli, but under low-sadness conditions this difference disappeared. The main reason is that the P1 amplitude evoked by global stimuli under low-sadness conditions was larger than under neutral or high-sadness conditions, a pattern that once again proved our inference. This was the direct ERP evidence that low sadness extended the scope of attention. That is to say, when participants felt a low level of sadness, they put more cognitive resources toward processing global stimuli at an early stage of visual perception. But as sadness became more intense, this phenomenon disappeared. One possible reason that the expansion of attention scope disappeared during high levels of sadness is that the emotions induced by high-sadness pictures were complex, including fear and aversion. This may have led to different physiological or psychological reactions from subjects, narrowing their attention range. The GFG group differed significantly from the LFG group, and the mean amplitude of the P1 component evoked by local stimuli was significantly greater than that evoked by global stimuli at all degrees of sadness. This indicated that sadness level did not affect scope of attention in participants who were biased toward processing global information.

We did not find the same pattern in the N2 and P3 components, which showed no significant difference between low-sadness and high-sadness conditions. Compared with local stimuli, global stimuli elicited greater N2 amplitudes in the LFG group. This probably indicated that participants experienced more interference during recognition of global stimuli in this experiment. The P3 component was the last component to be evoked before participant reaction occurred. We found only the sadness effect of P3 component in the LFG group, which showed that participants identified local stimuli and that a smaller P3 amplitude was evoked during high sadness compared with other levels of sadness. That is to say, higher sadness impaired the decision-making process in participants with local bias. However, there were no differences between the neutral and low-sadness conditions.

## Author Contributions

All authors listed have made a substantial contribution to the work. HW designed the experiment. HW and QZ wrote the first draft of the manuscript. YC and QZ finished revision of the manuscript. YC put forward some important suggestions and finished important test in the revision process of the manuscript. All authors have agreed to take HW and YC as the co-first author, and approved publication of this work.

## Conflict of Interest Statement

The authors declare that the research was conducted in the absence of any commercial or financial relationships that could be construed as a potential conflict of interest.
